# IGF-1 Antibody Prolongs the Effective Duration Time of Botulinum Toxin in Decreasing Muscle Strength

**DOI:** 10.3390/ijms14059051

**Published:** 2013-04-25

**Authors:** Lingjing Jin, Lizhen Pan, Wuchao Liu, Yan Guo, Yuguo Zheng, Qiang Guan, Zhiyu Nie

**Affiliations:** 1 Department of Neurology, Shanghai Tongji Hospital, Tongji University School of Medicine, Shanghai 200065, China; E-Mails: lizhen.pan@gmail.com (L.P.); lwcnh@163.com (W.L.); jas.123@163.com (Y.G.); guanqianglu@126.com (Q.G.); 2 Department of Biomedicine, China National Center for Biotechnology Development, Beijing 100036, China; E-Mail: zh@cncbd.org.cn

**Keywords:** botulinum toxin, insulin-like growth factor 1, muscle strength, muscle-specific receptor tyrosine kinase

## Abstract

Botulinum toxin type-A (Btx-A), a powerful therapeutic tool in various medical specialties, requires repeated injections to maintain its effect. Therefore, novel methods to prolong the effective duration time of Btx-A are highly needed. Rats were assigned to three major groups: control group (*n* = 30), Btx-A group (*n* = 30), and IGF-1 Ab groups. IGF-1 Ab groups were composed by sub-groups A1–A5 (each has 25 rats) for the subsequent IGF-1Ab dose-effect study. Muscle strength was determined by a survey system for rat lower limbs nerve and muscle function. Muscle-specific receptor tyrosine kinase (MuSK), Insulin-like growth factor binding protein-5 (IGFBP5), and growth-associated protein, 43-kDa (GAP43) were determined by real-time polymerase chain reactions (PCRs) and Western blot. We found that Btx-A decreased the muscle strength, with a paralysis maintained for 70 days. IGF-1Ab prolonged the effective duration time of Btx-A. Real-time PCRs and Western blot showed that IGF-1Ab delayed the increase of MuSK and IGFBP5 after Btx-A injection, without affecting GAP43. These results indicate that IGF-1Ab might prolong the effective duration time of Btx-A on muscle strength through delaying the increase of MuSK. It would be interesting to determine whether IGF-1Ab can be used as an auxiliary measure to the Btx-A treatment in the future.

## 1. Introduction

Botulinum toxins (BTs), the exotoxin of the obligate anaerobe Clostridium botulinum, are comprised of a family of seven serotypes (A–G) that block the transmission at neuromuscular junctions (NMJs) by inhibiting quantal acetylcholine (Ach) release [1]. Since its first use as a therapy for strabismus in 1981 [2], BT has become a powerful therapeutic tool in multiple medical specialties including neuromuscular, ophthalmological, gastrointestinal, urological, orthopaedic, dermatological, secretory, painful and cosmetic disorders [3]. Among BTs, Type A botulinum toxin (Btx-A) is a well-established and licensed one for clinical therapeutics.

As the selective blocking effect of Btx-A usually wears off 3–4 months after muscle tissue injection [4], repeated injections are usually needed to maintain its desired effect. It is well-known that repetitive injection can trigger immune responses and develop resistance to the toxin as well [5]. Thus, novel methods to prolong the effective duration time of Btx-A are highly needed.

After the injection of BTs, loss of innervation induces sprouting of motor nerve terminals. These sprouts are the sole synaptic structures undergoing exoendocytosis and are responsible for functional recovery at the onset of repair of nerve-induced muscle twitching [6]. A significant increase in the neuromuscular junction quantification decreases the effects of Btx-A [7]. Nerve sprouts after Btx-A injection are accompanied by the accumulation of insulin-like growth factor 1 (IGF-1) [8], which is synthesized by motoneurons and skeletal muscle [9]. IGF-1 signaling is involved in muscle development and regeneration [10], neuromuscular junction formation and preservation of morphology of postsynaptic NMJs [11,12], neurite sprouting of motoneurons and the elongation of the regenerating axons [13]. Although IGF-1 is shown to promote nerve sprouting and sprouts are important for the recovery of NMJs, it remains unclear whether IGF-1Ab could prevent the recovery of NMJs and prolong the effect of Btx-A.

## 2. Results

### 2.1. Btx-A Decreases the Muscle Strength, with a Paralysis Maintained till 70 Days

**Figure 1:**
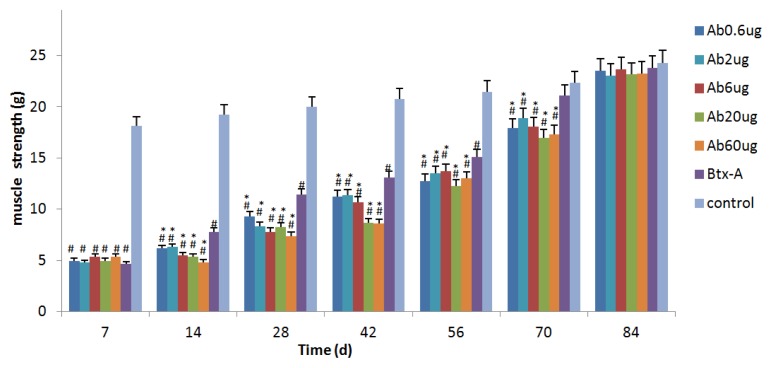
IGF-1Ab prolongs the effective duration time of Btx-A. Muscle strength was determined by a survey system for rat low limbs nerve and muscle function at different times. *p < 0.05, *versus* Btx-A group. # *p* < 0.05, *versus* control group.

As shown in Figure 1, a single injection of 0.5 unit Btx-A decreased the muscle strength generated in the botulinum toxic treated limb, which reached peak paralysis on day 7 (4.66 ± 0.59 g) and began to increase on day 14. The muscle strength in Btx-A group remained significantly lower than control until day 56 (*p* < 0.05). Paresis was maintained until strength returned to control levels by 70 days without causing death and muscle.

### 2.2. IGF-1Ab Prolongs the Effective Duration Time of Btx-A

In subgroups A1–A5, the recombinant IGF-1Ab with the dosages of 0.6 μg, 2 μg, 6 μg, 20 μg, 60 μg were intramuscularly injected to gastrocnemius respectively on day 3. As indicated in Figure 1, no significant difference of muscle strength was observed between the group Btx-A and each subgroup A on day 7. On day 14, each subgroups showed decreased strength compared with the Btx-A group. Muscle strength in subgroups was statistically lower than the Btx-A group for 70 days (*p* < 0.05). On day 84, the muscle strength of the subgroups A1–A5 also recovered. These data indicates that IGF-1Ab prolongs the effective duration time of Btx-A.

**Figure 2:**
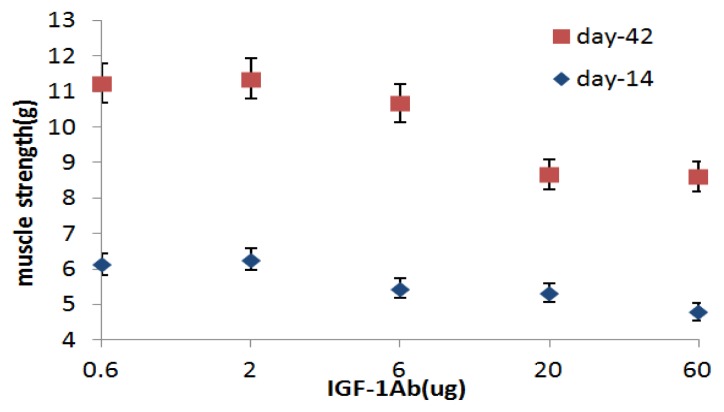
A dose-dependent effect of IGF-1Ab in maintaining the decreased muscle strength caused by Btx-A on day 14 and day 42.

As shown in Figure 2, IGF-1Ab shows a dose-dependent effect in maintaining the decreased muscle strength caused by Btx-A. On day 14, muscle generated in subgroups A3–A5 was lower than subgroup A1–A2 (*p* < 0.05) and there was no statistically difference among subgroups A3–A5 (*p* > 0.05). After that, muscle strength began to increase, on day 42, muscle strength in subgroups A4–A5 was still less than subgroups A1–A2, but in subgroup A3 it was not different from subgroups A1–A2 (*p* > 0.05). This time course study at the five dosages of IGF-1Ab indicates that the dosage of 20ug is the threshold dose. Therefore, this dosage was selected in the subsequent studies.

**Figure 3:**
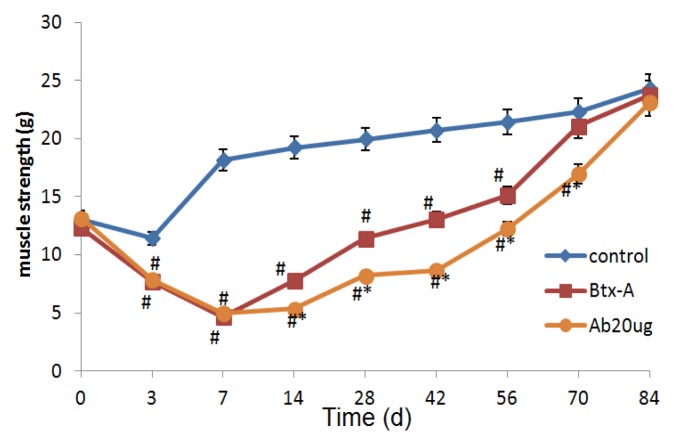
The paralysis effects of Btx-A last longer after IGF-1Ab injection. * *p* < 0.05, compared to the Btx-A group. # *p* < 0.05, compared to the control group.

Moreover, as shown in Figure 3, on day 14, though muscle strength began to increase in subgroup A4, it was still lower compared with Btx-A group (*p* < 0.05) and this effect remained until day 70. On day 70, the values in Btx-A group were similar to the controls, but the values from subgroup A4 were lower than Btx-A group and controls till day 84. These results suggest that the paralysis effect of Btx-A lasts longer after IGF-1Ab injection.

### 2.3. IGF-1Ab Delays the Increase of MuSK and IGFBP5 after Btx-A Injection

**Figure 4:**
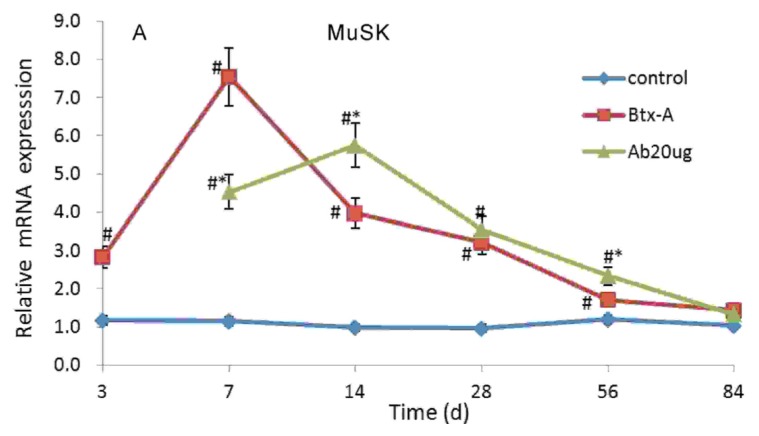

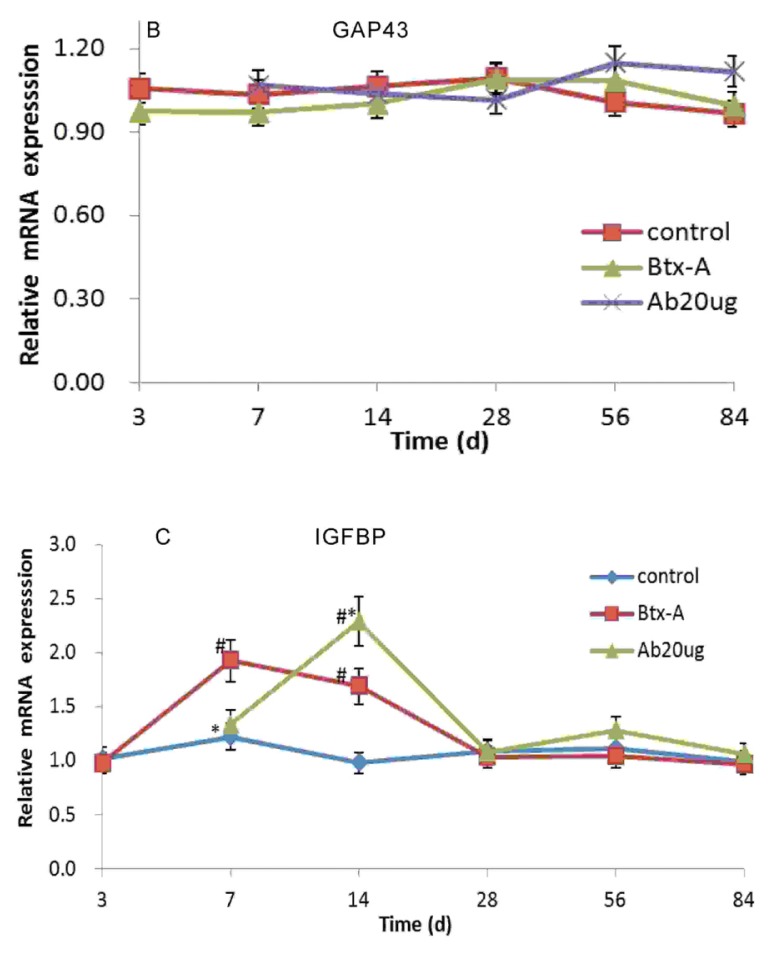
IGF-1Ab delays the increase of MuSK and IGFBP5 without affecting GAP43 at mRNA level after Btx-A injection. (**A**) The mRNA expression level of MuSK; (**B**) The mRNA expression level of GAP43; (**C**) The mRNA expression level of IGFBP5. * *p* < 0.05, compared to the Btx-A group. # *p* < 0.05, compared to the control group.

**Figure 5:**
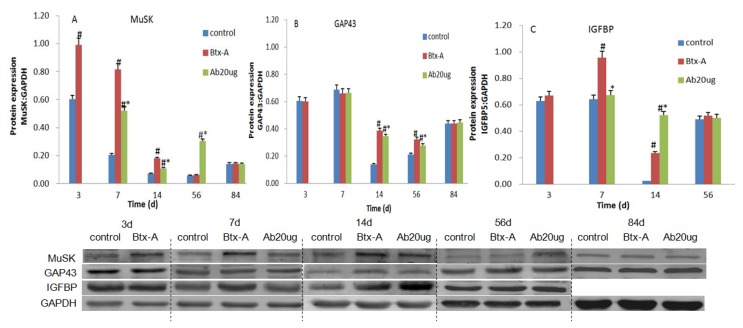
IGF-1Ab delays the increase of MuSK and IGFBP5 without affecting GAP43 at protein level after Btx-A injection. (**A**) Protein expression of MuSK; (**B**) Protein expression of GAP43; (**C**) Protein expression of IGFBP5. Representative bands from the Western blot are shown below the summary graph. * *p* < 0.05, compared to the Btx-A group. # *p* < 0.05, compared to the control group.

The mRNA expression level of MuSK in skeletal muscle increased significantly 3 days after Btx-A injection, and peaked on day 7. Following IGF-1Ab injection on day 3, the gene expression upregulated on day 7, peaked on day 14 (Figure 4A). The mRNA expression level of IGFBP5 increased on day 7 and then peaked off. After IGF-1Ab treatment, the gene expression level of IGFBP5, which did not increase on day 7 compared with the group control and was lower than Btx-A group (*p* < 0.05), peaked on day 14 (Figure 4C). Similar changes were observed for MuSK and IGFBP5 at the protein level (Figure 5A,C). However, GAP43 did not significantly change following Btx-A and IGF-1Ab injection both at the mRNA and protein level (Figures 4B and 5B).

## 3. Discussion

The major findings of this study are as follows. Firstly, Btx-A decreases the muscle strength, with a paresis maintained for 70 days. Secondly, IGF-1Ab prolongs the effective duration time of Btx-A. Finally, IGF-1Ab delays the increase of MuSK and IGFBP5 after Btx-A injection. Other laboratories have investigated altering the IGF-1 pathway with antibodies. In the study of Caroni *et al*., they demonstrated that local delivery of IGF-1 Ab in Botulinum toxin A-paralyzed skeletal muscle effectively prevented nerve sprouting in that muscle [14]. In the report by Harrison *et al.*, they found that treatment with antibody to IGF-I receptor prevented the increase in neuromuscular junction [15]. However, none of them have explored the change of muscle strength directly after the pathway of IGF-1 was blocked. To the best of our knowledge, this is the first report showing IGF-1Ab prolongs the effective duration time of Btx-A on the muscle strength.

Several ways have been tried to increase the duration and effectiveness of Btx-A, including high-volume preparation, higher dosages, electrical stimulation post-injection, intramuscular placement (ultrasound techniques or electromyography), repeated injection [16–19]. However, total dosages and inject volumes may be limited by adverse events [18]. Immunoresistance due to antigenicity of the toxin may also induce treatment failure, and the risk of developing antibody-induced treatment failure has been shown to be increased with short injection intervals, repeated injection and high injected doses [20]. Moreover, no definite evidence have been presented that the adjuvant electrical stimulation post-injection was superior to Btx-A alone in alleviating spasticity in terms of both electrophysiological and clinical assessment [21]. Recently, a novel recombinant protein, Targeted Secretion Inhibitor (TSI), has already progressed in the clinical applications. However, as TSIs are innovative therapeutic proteins, many questions have not been well solved yet [22]. Therefore, novel methods to prolong the effective duration time of Btx-A are desirable.

IGF-1 exerts growth-promoting effect on nerve outgrowth. Introduction of a non-viral vector containing human IGF-1 into paralyzed rat larynx increased the number of neuromuscular junctions and prevented denervation-induced muscle atrophy [23]. Neutralizing antibodies to IGF-1, when focally applied, resulted in reduction of terminal axon sprouting of injured facial nerve [24]. In the present study, we provided IGF-1Ab as a novel method to prolong the effective duration time of Btx-A on the muscle strength. This phenomenon also provides indirect evidence that IGF-1 plays an important role in the recovery of NMJ.

GAP43 is a neurotrophic factor synthesized in presynaptic axons and is involved in neuronal development [25]. MuSK is the receptor of proteoglycan agrin, which orchestrates nicotinic acetylcholine receptor (nAChR) clustering, and is also essential for neuromuscular synapse formation of postsynaptic apparatus [26]. MuSK expression is patterned in independent of innervation and ectopic MuSK expression is sufficient to promote ectopic motor axon growth and stimulate synapse formation [27]. It is well-known that Musk is required for clustering AChRs in the central region of the nerve-independent muscle patterning [28]. The nerve terminal sprouting proceeds only after postsynaptic specializations have formed and induced the new NMJ [29]. In our study, MuSK upregulated on day 3 and peaked on day 7, then muscle strength begain to recovery. IGFBP-5 is the predominant high-affinity IGFBPs secreted by skeletal muscles [30]. IGFBP-5 is primarily an inhibitor of IGF actions in cultured skeletal muscle cells [31] and it has IGF-independent effects function as carrier proteins in circulation [32]. In the present study, we found that IGF-1Ab delayed the increase of MuSK and IGFBP5 after Btx-A injection and influenced the recovery process, but did not change GAP43. These results indicate that IGF-1Ab might prolong the effective duration time of Btx-A on the muscle strength through delaying the increase of MuSK.

## 4. Experimental Section

The animals used in this study were maintained in accordance with the Guide for Care and Use of Laboratory Animals published by the US National Institutes of Health (NIH Publication No. 85–23, revised 1996) and the Policy of Animal Care and Use Committee of Tongji University.

### 4.1. Intervention Protocols

One hundred and eighty-five male Sprague-Dawley rats, weighing 185–215 g, from B & K Universal Group Limited (Shanghai, China) were used. Rats were assigned to three major groups: control group (*n* = 30), Btx-A group (*n* = 30), and IGF-1 Ab groups. IGF-1 Ab groups were composed by sub-groups A1–A5 (each has 25 rats) for the subsequent IGF-1Ab dose-effect study.

A vial of lyophilized Btx-A (Botox®, Allergan, Co. Mayo, Ireland) was reconstituted with sterile saline solution in a concentration of 5 units/mL. In Btx-A group and IGF-1 Ab group, a volume of 0.1 mL Btx-A was injected into a site in the right gastrocnemius muscle, and the time point of injection was considered day 0 (d0). On day 3, the recombinant IGF-1Ab (Boster-engineering Co. Led, Wuhan, China) was dissolved in phosphate-buffered saline (PBS) containing 0.1% bovine serum albumin (BSA). Equal volumes (0.1 mL) of IGF-1Ab with a dosage of 0.6 μg, 2 μg, 6 μg, 20 μg, 60 μg were intramuscularly injected to group A1–A5 respectively. Controls received an equivalent volume of saline injections in the right gastrocnemius muscle.

### 4.2. Determination of Muscle Strength

To evaluate the muscle strength of right hind limb, rats were lightly anesthetized with an intraperitoneal injection of pentobarbital (30 mg/kg; Tongji hospital Laboratory) and were secured on a special adjustable operating table (CN202036227U) on day 0, day 3, day 7, day 14, day 28, day 42, day 56, day 70 and day 84. The operating table can fix the rat while keeping the forward mobility of the ankle joint. A survey system (CN102599921A) which comprises fixing device, sensing means and data handling equipment were used in this study for the evaluation of nerve and muscle function of rat limbs. Rats were lightly anesthetized and fixed on the operating table (CN202036227U). When sciatic nerve is stimulated, the contraction of gastrocnemius leads to plantar flexion and rotates a footboard, which would be converted to electrical signals through tonotransducer and recorded by the computer. With this instrument, the change of muscle strength induced by 0.1 μ Btx-A could be detected. Similar methods have been used by other researchers to assess the effects of Btx-A [33]. This system allows the assessment of biological activity of Btx-A via measuring muscle strength generation and the effective duration time as well. Transcutaneous electrodes were placed near the sciatic nerve. To expose the sites for electrode placement, hair was gently depilated from the hind quarter of the leg by depilatory paste. The sciatic nerve was stimulated (28 V over 0.4 ms) and the muscle strength generated by the gastrocnemius was amplified and recorded using data handling equipment.

### 4.3. Real-Time Polymerase Chain Reactions (PCRs)

The right gastrocnemius muscle samples were harvested on day 3, day 7, day 14, day 28, day 42, day 56, day 84 from the control group and Btx-A group. As for the IGF-1 Ab subgroups, the samples were harvested from day 7. At each time point, 5 rats were sacrificed by cervical dislocation from each group. Tissue was snap frozen in liquid nitrogen and stored at −80 °C until analysis.

Total RNA was extracted by Trizol (Invitrogen, Carlsbad, CA, USA). The prepared total RNA was reverse-transcribed by PrimeSctipt RT Reagent Kit (TaKaRa, Dalian, China). cDNA was used as the template for the subsequent real-time PCR analysis. Real-time PCR assays were performed using a Rotor-gene 3000 real-time PCR detection system (Corbett Research, Sydney, Australia) in a 20 μL final reaction volume containing 10 μL SYBR Premix Ex taq (TaKaRa), 0.4 μL forward primer (10 μmol/L), 0.4 μL reverse prime (10 μmol/L), 1 μL cDNA template at a final concentration of 50 ng and 8.2 μL ddH_2_O. A quantification cycling protocol was: 95 °C for 10 min, followed by 40 cycles at 95 °C for 5 s, 60 °C for 15 s and 72 °C for 20 s. Experiments were performed with triplicates for each sample. To correct for potential variances between samples in mRNA extraction or in reverse transcribed efficiency, the mRNA content of each gene was normalized to the expression of β-actin within the same sample. Sequences for all PCR primers are as follows: forward 5′-TTACTGCCCTGGCTCCTA-3′ and reverse 5′-ACTCATCGTACTCCTGCTTG-3′ for β-actin; forward 5′-GACACCCGCTACAGCATCCG-3′ and reverse 5′-CACCGCTCCTCCCACTCCAT-3′ for Muscle-specific receptor tyrosine kinase (MuSK); forward 5′-GTGGCCGCAAACGTGGCATCT-3′ and reverse 5′-GTCGAAGGCGTGGCACTGAA-3′ for IGF binding protein-5 (IGFBP5); forward 5′-CTAAACAAGCCGATGTGCCT-3′ and reverse 5′-CTTCTTTACCCTCATCCTGTCG-3′ for Growth-associated protein, 43-kDa (GAP43).

### 4.4. Western Blot

The gastrocnemius muscle samples were harvested on day 3, day 7, day 14, day 56, day 84 and homogenised in cold radioimmunoprecipitation assay lysis buffer (Beyotime, China) plus 1:100 volume of PMSF. After centrifugation at 12,000× *g* at 4 °C for 5 min, protein content was assayed in duplicate by the BCA protein assay kit (Pierce, Rockford, IL, USA). Protein samples (20 μg/lane) were separated by SDS-PAGE and transferred to PVDF membranes (Millipore, Billerica, MA, USA). Blots were blocked in 5% (*w*/*v*) BSA (Sigma, Santa Clara, CA, USA) and PBS/0.1% Tween-20 solution at room temperature for 1 h, and then incubated with rabbit anti-rat primary antibodies (Abcam, Cambridge, MA, USA) at the following dilutions: anti-IGFBP5 (1:1000), anti-MuSK (1:100), anti-Gap43 (1:1000) at 4 °C overnight. Blots were then incubated with IRDye800-conjugated secondary antibody (Rockland, Gilbertsville, PA, USA) at 1:8000 for 1 h at room temperature. Images were acquired using a Li-COR Odyssey Infrared Imaging System (Li-COR Biosciences, Lincoln, NE, USA) and intensity for each band was quantified. GAPDH was used as a loading control.

### 4.5. Statistical Analysis

Data are expressed as the Mean ± SD. A one-way analyses of variance (ANOVA) was conducted to evaluate the data. If a significant difference was found, a Student-Newman-Keuls multiple comparison test was conducted to determine which group differed significantly. *p*-values less than 0.05 were considered statistically significant.

## 5. Conclusions

The present study has provided direct evidence that intramuscular injection of IGF-1Ab can enhance and prolong the muscle paralysis of Btx-A. IGF-1Ab might prolong the effective duration time of Btx-A on the muscle strength through delaying the increase of MuSK. It would be interesting to determine whether IGF-1Ab can be used as an auxiliary measure to the Btx-A treatment in the future.
